# Survey and Molecular Characterization of Trichomonads in Pigs in Anhui Province, East China, 2014

**Published:** 2018

**Authors:** Wen-Chao LI, Kai WANG, Yan LI, Li-Ping ZHAO, Yi XIAO, You-Fang GU

**Affiliations:** College of Animal Science, Anhui Science and Technology University, Fengyang, 233100, China

**Keywords:** *Tritrichomonas suis*, *Tetratrichomonas buttreyi*, *Pentatrichomonas hominis*, Pigs, China

## Abstract

**Background:**

In pigs, several different trichomonad species such as *Tritrichomonas foetus*, *Tetratrichomonas buttreyi,* and *Pentatrichomonas hominis* have been described as inhabiting the digestive tract. However, little information is available on the epidemiology of these neglected parasites in the Chinese pig population.

**Methods:**

The prevalence of *T. suis*, *T. buttreyi* and *P. hominis* among 500 fecal specimens from pigs at seven pigs farms in Anhui Province in China between Oct and Dec 2014, was determined by PCR and DNA sequence analysis of the small subunit ribosomal RNA (SSU rRNA) genes.

**Results:**

The prevalence rates for *T. suis*, *T. buttreyi,* and *P. hominis* were 2.8% (14/500), 42.0% (210/500) and 7.8% (39/500), respectively. Mixed infections of two or three trichomonads were detected in 24 samples. The prevalence of the three trichomonads differed significantly between some age groups, with higher infection rates of *T. suis* and *T. buttreyi* in nursery pigs and *P. hominis* in preweaned pigs. The SSU rRNA sequences from *T. suis* and *P. hominis* showed 100% homology with their respective homologous database sequences. However, we observed minor allelic variations in the SSU rRNA sequences from *T. buttreyi*, and the five representative sequences identified were named firstly as types 1, 2, 3, 4 and 5. Moreover, type 1 was found to be dominant in the present study.

**Conclusion:**

These findings highlight the potential risk posed by pigs in the transmission of trichomonad infections to humans and other animals.

## Introduction

Parasites of the *Tritrichomonas* genus, which consists of commensal and pathogenic species from a broad host range of vertebrates and invertebrates, are frequently encountered in veterinary medicine (1–4). They are characterized by the presence of three-to-five anterior flagella and a single recurrent flagellum that functions as an undulating membrane (5). The following three different trichomonad species were originally reported as pathogenic protists in pigs: *Tritrichomonas suis*, *T. rotunda*, and *Tetratrichomonas buttreyi* (6). *Hypotrichomonas acosta* and *Trichomitus batrachorum*, previously isolated from squamate reptiles and a frog, respectively, have also been described in pigs (7). Recently, our laboratory reported the occurrence of *Pentatrichomonas hominis* in pigs and 24.1% (38/158) of the pigs in Changchun, China tested positive for this species (4, 8).

Among the above mentioned porcine trichomonad species, *T. suis* is the well-studied and has been reclassified as a synonym of the parasitic trichomonad responsible for bovine and feline trichomonosis, *T. foetus* (9–11). *T. suis* was initially regarded commensalic and nonpathogenic in pigs (12). However, *T. suis* was a facultative pathogen of the pig large intestine, thus highlighting its medical importance (9–10, 13). *T. buttreyi* is found in pig and cattle fecal samples and is considered a commensal organism (10, 14). Similarly, *P. hominis* is presumed to be a commensal organism that can overgrow opportunistically in hosts with diarrhea from other causes (2). However, *P. hominis* was described as a pathogen associated with gastrointestinal discomfort, respiratory tract infections and rheumatoid arthritis in humans (15–17). Additionally, *P. hominis* has the ability to propagate in the cecum of piglets and is most likely a pathogen in pigs with underlying comorbidities (4).

China is the world’s largest producer and consumer of pork. Pig farming plays a particularly important role in animal husbandry in Anhui Province. The rapid growth in pig farming and the high human population density in this region may increase the spread of infectious micro-organisms carried in the feces of domestic animals (including trichomonads) to humans. Unfortunately, few published studies are available on the epidemiology of trichomonads in pigs in China. Recently, our laboratory reported the prevalence of trichomonads in pigs in Changchun city, Jilin Province, northeast China (8).

To date, no studies have been published for pigs in Anhui Province, China. In the present study, we conducted an extensive survey of pigs and accurately determined the prevalence of trichomonads using molecular methods. We also genetically characterized the trichomonads identified in this study and discussed the risk factors for trichomonad infections in pigs in Anhui Province, China.

## Materials and Methods

### Fecal specimen collections

**Fig. 1:**
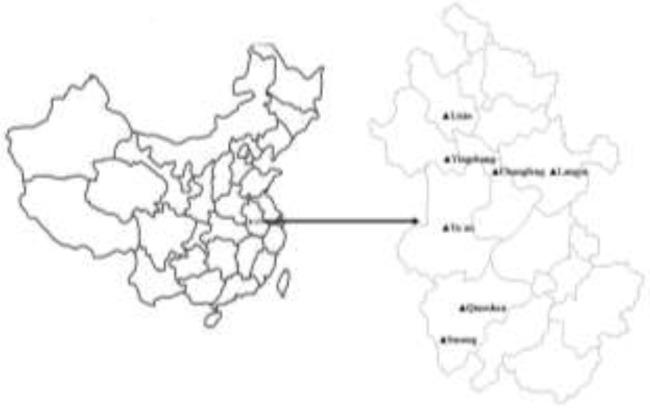
Location of the sampling areas

Overall, 500 fresh fecal samples including those from preweaned pigs (under 30 d old), nursery pigs (30–60 d old), and growing pigs (over 60 d old) were collected from seven pig farms located in seven prefectures in Anhui Province between Oct and Dec 2014 (Fig. 1). All the fecal samples were stored at 4 °C prior to DNA extraction within three days.

The present experimental protocol was conducted in accordance with the strict guidelines and recommendations from the Guide for the Animal Care and Welfare Committee of Anhui Science and Technology University, China.

### DNA extraction

Genomic DNA was extracted directly from a 200-mg sample of each fecal specimen using a Stool DNA Kit (Tiangen, Beijing, China), according to the manufacturer’s instructions. The DNA was eluted in a 50 μl volume and stored at −20 °C until use.

### PCR analysis of three trichomonad species

Amplification of the partial SSU rRNA genes from *T. suis*, *T. buttreyi* and *P. hominis* was performed (4). Briefly, trichomonad genus detection was based on a primary PCR with forward primer FF (5′-GCGCCTGAGAGATAGCGACTA-3′) and reverse primer RR (5′-GGACCTGTTATTGCTACCCTCTTC-3′). Amplification, in a final volume of 20 μl, involved heating the reactions at 95 °C for 10 min, followed by 30 cycles of 95 °C for 1 min, 60 °C for 1 min, 72 °C for 1 min 30 sec and a final extension at 72 °C for 10 min. The primary PCR products were subjected to species-specific trichomonad characterization using a secondary PCR with the following primers: sF (5′-GGTTGTTTGTATAGGATTGC-3′) and sR (5′-TGCCCTCATAAAAGGACAA-3′) for *T. suis* (451 bp); bF (5′-GTTTTTTCTCAGGCAGCAATG-3′) and bR (5′-GCAACCTAGAAACCTAGGCG-3′) for *T. buttreyi* (623 bp); and hF (5′-TGTAAACGATGCCGACAGAG-3′) and hR (5′-CAACACTGAAGCCAATGCGAG G-3′) for *P. hominis* (339 bp).

The concentrations and reagents used for the secondary amplification were identical to those used in the primary PCR, except that 2 μl of the primary PCR product was substituted for genomic DNA.

The secondary PCR was also amplified according to the conditions except that the denaturation duration (45 sec instead of 30 sec) and extension duration (45 sec instead of 30 sec) for *T. suis* and *P. hominis* were modified (4). All PCRs were performed in duplicate. Control samples without DNA (negative control) and with the three trichomonad species genomic DNA (positive control) were included in each PCR run.

### DNA sequencing

Secondary PCR products were subjected to electrophoresis in 2% agarose gels and then visualized by ethidium bromide staining. PCR products were purified and the accuracy of the nucleotide sequences was confirmed by two-directional sequencing. DNA sequences were aligned against reference sequences using BioEdit v 7.1.3.0 software (Ibis Biosciences, Carlsbad, CA, USA). Neighbor-joining trees were constructed using MEGA 5 software (http://www.megasoftware.net/), and evolutionary distances were calculated using the Kimura 2-parameter model. The reliability of the cluster formation was evaluated by the bootstrap method with 1000 replicates. Representative SSU rRNA sequences from this study have been deposited in GenBank under accession number KX833160 (for *T. suis*), KX833155–KX833159 (for *T. buttreyi*), and KX833161 (for *P. hominis*).

### Statistical analysis

The χ^2^ test was used to compare the three trichomonad infection rates in the different groups of pigs, using SPSS for Windows (13.0 standard version; (Chicago, IL, USA). Differences were considered statistically significant at *P*<0.05.

## Results

### Prevalence of trichomonad infections in pigs

Of the 500 samples tested, 237 pigs (47.4%) were positive for one-to-three known Trichomonadida order members. *T. buttreyi* infections (42.0%) was predominated, followed by *P. hominis* (7.8%) and *T. suis* (2.8%). Four (0.8%) contained only *T. suis*, 186 (37.2%) contained only *T. buttreyi*, and 23 (4.6%) contained only *P. hominis*. Double infections with *T. suis* and *T. Buttreyi*, and with *T. buttreyi* and *P. hominis* were found in eight (1.6%) and 14 (2.8%) samples, respectively. Mixed infections with all three trichomonads were observed in two (10.4 %) samples (Table 1).

**Table 1:** Prevalence of trichomonad infections in pigs in Anhui Province

***Location (s)***	***Samples (N)***	***T. suis only Positives (%)***	***T. buttreyi only Positives(%)***	***P. hominis only Positives (%)***	***T. suis + T. buttreyi Positives (%)***	***T. suis + P. hominis Positives (%)***	***T. buttreyi+ P.hominis Positives (%)***	***T. suis + T. buttreyi+ P. hominis Positives (%)***
Lixin	160	2(1.3)	72(45.0)	7(4.4)	7(4.4)	0	10(6.3)	2(1.3)
Susong	33	0	7(21.2)	5(15.2)	0	0	3(9.1)	0
Qianshan	50	0	26(52.0)	0	0	0	0	0
Langya	64	0	4(6.3)	5(7.8)	0	0	0	0
Changfeng	40	0	0	3(7.5)	0	0	0	0
Yingshang	130	2(1.5)	67(51.5)	3(2.3)	1(0.8)	0	1(0.8)	0
Yu an	23	0	10(43.5)	0	0	0	0	0
Total	500	4(0.8)	186(37.2)	23(4.6)	8(1.6)	0	14(2.8)	2(0.4)

Infections with *T. suis* were only found in Lixin and Yingshang and the prevalence rates were 6.9% (11/160) and 2.3% (3/130), respectively. Lixin recorded the highest prevalence of *T. buttreyi* (56.9%; 91/160), followed by Yingshang (53.1%; 69/130), Qianshan (52.0%; 26/50), Yuan (43.5%; 10/23), Susong (30.3%; 10/33), and Langya (6.3%; 4/64). For *P. hominis*, the highest prevalence was detected in Susong (24.2%; 8/33), followed by Lixin (11.9%; 19/160), Langya (7.8%; 5/64), Changfeng (7.5%; 3/40), and Yingshang (3.1%; 4/130) (Table 1).

### Age distributions of the porcine trichomonad infections

The *T. suis* infection rate (8.9%; 9/101) in nursey pigs was significantly higher than that of the preweaned pigs (0.6%, 1/168; *P*<0.01) and growing pigs (1.7%; 4/231; *P*<0.01), but the difference in the infection rates between these groups was not significant (*P*>0.05). For *T. buttreyi*, the nursery pigs had an infection rate (77.2%; 78/101) significantly higher than that of the preweaned pigs (26.8%, 45/168; *P*<0.01) and growing pigs (37.7%, 87/231; *P*<0.01). Furthermore, the infection rate difference between the preweaned and growing pigs was also significant (*P*<0.05). For *P. hominis*, the preweaned pigs had an infection rate (11.9%, 20/168) significantly higher than that of the nursery pigs (4.00%, 4/101; *P*<0.05), but the difference in the infection rates were not significant between these two groups or any other two groups (data not shown).

### Molecular characterization of the trichomonad isolates

**Fig. 2:**
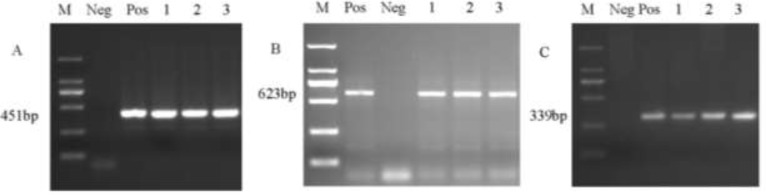
Analysis of nested PCRs of *T. suis*, *T. buttreyi*, and *P. hominis* with 1% agarose gel electrophoresis, shown on panel A-C respectively. M: DL2000 DNA marker, 1 to 3: pigs fecal samples, Neg: negative control, Pos: positive control

Nested PCR resulted in three specific bands of approximately 452 bp (*T. suis*), 623 bp (*T. buttreyi*), and 339 bp (*P. hominis*) (Fig. 2).

The *T. suis* nucleotide sequences from the present study showed 100% homology with the reference sequences from different hosts including pig (KM205209), *Bos taurus* (AY055799), *Sus scrofa* (AY055800), and *Felis catus* (AF466749). Similarly, the *P. hominis* sequences from this study showed 100% homology with the reference sequences from different hosts including pig (KM205209), dog (KC953860), *Felis catus* (KC594038), *Canis lupus* (KC953860), and *Ovis aries* (JX565028).

**Fig. 3:**
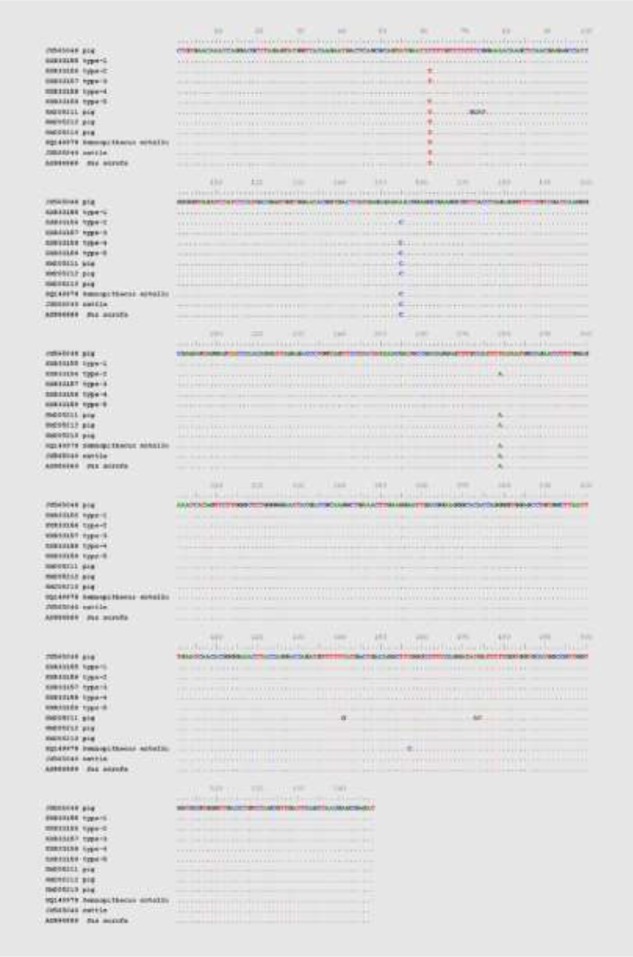
Alignment of representative SSU rRNA sequences from the *T. buttreyi* isolates obtained in this study and those of *T. buttreyi* isolates from other studies. Only nucleotide sequences that differ from the reference sequence (JX565048) are indicated. Dots represent the consensus sequence of all the *T. buttreyi* strains were shown

Among the *T. buttreyi*-positive samples, the 200 of them successfully sequenced exhibited 99.5%–99.8% sequence identity. The five different sequences observable in the alignment of the 200 sequences from this study only differed at three variable positions (Fig. 3).

**Fig. 4:**
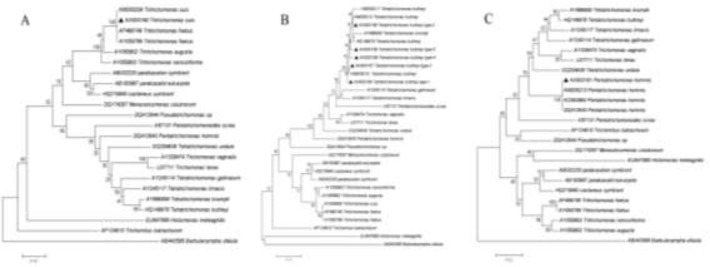
Phylogenetic relationships of the SSU rRNA nucleotide sequences from trichomonas isolates. Phylo-genetic trees for *T. suis*, *T. buttreyi*, and *P. hominis* are shown in panels A–C, respectively. The trichomonad species isolates obtained in this study are indicated by triangles before their names.

These five representative sequences can be classified into five sequence types referred to as types 1, 2, 3, 4 and 5. Out of the 200 sequences, 102 (51.0%), 60 (30.00%), 22 (11.0%), 5 (2.5%), and 11 (5.5%) separated into types 1, 2, 3, 4 and 5, respectively. In the common part of our alignment (548 positions), the sequence types 1, 2, 3, 4 and 5 also share 98.2%–100% identity with the homologous sequences from *T. buttreyi* from other hosts, including cattle, *S. scrofas* and *Semnopithecus entellus* (Fig. 2). The neighbor-joining trees confirmed without doubt that the sequences obtained in this study belong to *T. suis*, *T. buttreyi*, and *P. hominis* (Fig. 4).

## Discussion

Traditionally, trichomonad infections are diagnosed through microscopic examination of fresh fecal samples or cultures of fecal samples. However, very often different trichomonad species that share similar morphological features cannot be differentiated by microscopic examination (18, 19). Therefore, sensitive and specific molecular methods are now used widely to identify trichomonad species and strains (20–22). Although several porcine trichomonad species have been described, only a handful of epidemiological reports documenting naturally occurring infections of pigs with trichomonads around the world have been published, partly because of the relative lack of importance of these species in veterinary medicine (8, 23–25). In the present study, the prevalence of the three trichomonad species in pigs in Anhui, East China, was investigated by nested PCR targeting the SSU rRNA gene and genetic similarities among the three trichomonads in pigs and other hosts were also analyzed. We found that the overall infection rate for trichomonad species in the pigs, as determined by PCR, was 47.4% (237/500). This proportion is quite similar to the value (43.0%; 68/158) for northeast China (8), but lower than the value (52.1%; 100/192) from in *situ* hybridization and the value (77.4%; 24/31) from culture experiments (25).

Besides, six trichomonad species have been identified in pigs, namely, *T. suis*, *T. rotunda*, and *T. buttreyi*, *H. acosta*, *T. batrachorum* and *P. hominis* (4, 6–8). Among these species, *T. suis* is the most common in various geographical locations of the world (25% in UK, 56.3% in Japan, 12.0% in China, 64.5% in Austria). In contrast, our study found a low prevalence (2.8%) of *T. suis* in pigs in East China. Indeed, we found that *T. buttreyi* (42.0%) was the most common species in the present study.

These results clearly differ from those reported for northeast China, where *P. hominis* (24.1 %) was predominant, followed by *T. buttreyi* (14.6%) and *T. suis* (12.0%) (8). The differing prevalences of the trichomonad species from the various studies may reflect possible variations in the samples and in the examination methods used, as well as age, sample sizes, seasonality, host health status, specimen collection timing, and geo-ecological conditions (26).

In the present study, all the age groups were positive for three trichomonads, which is consistent with the results of a previous study in northeast China (8). Nevertheless, an age-related difference was observed in the prevalence of the three trichomonads in this study. *T. suis* and *T. buttreyi* had significantly higher prevalence rates in nursery pigs than in preweaned and growing pigs (*P*<0.01), whereas preweaned pigs had a significantly higher infection rate for *P. hominis* than that of the nursery pigs (*P*<0.05). Contrastingly, no significant relationship was found for the presence of the three parasite species and the animal age in previous studies conducted in northeast China (8). The SSU rRNA sequences obtained from *T. suis* and *P. hominis* our study were 100% identical to the sequences obtained from other animal isolates, which is consistent with previous observations (8).

Interestingly, most *T. suis* isolates from pigs are the ‘cattle genotype’, while a few of them are the ‘cat genotype’, as determined mainly by the presence of a conserved single nucleotide polymorphism in the internal transcribed spacer-2 gene (24, 25). In the present study, the genotypes of the *T. suis* isolates from pigs remain unresolved, and additional experimental studies are needed to obtain better genotyping.

Our study has also described minor allelic variation in *T. buttreyi* from East China, a result similar to that of a previous study in northeast China (8). The five representative sequences from the present study contained only three variable positions but allowed the identification of five sequence types (types 1 to 5). The minor genetic distinctions between the five types indicated that they were from the same *T. buttreyi* strain. Some sequences from different hosts currently available in GenBank displayed 100% identity to types 1 to 3 in this work, respectively, and the majority of them belonged to the type 2 classification according to our BLAST check and comparative analysis. Moreover, type 1 (51.0%) was found to be dominant in the present study, followed by type 2 (30.0%) and type 3 (11.0%), with a few pigs also infected with types 4 and 5.

## Conclusion

Pigs can act as potential sources of trichomonad infections in humans and other animals. Because of the high frequency of human contact with pigs in China, further studies are needed to clarify the significance of pigs in the epidemiology of trichomonads in humans and other animals.
